# Risk of surgical site infection after hip hemiarthroplasty of femoral neck fractures: a systematic review and meta-analysis

**DOI:** 10.1007/s00402-024-05384-5

**Published:** 2024-05-28

**Authors:** Ubong Silas, Christof Berberich, Priscilla Anyimiah, Dominik Szymski, Markus Rupp

**Affiliations:** 1 Coreva Scientific GmbH & Co. KG, Koenigswinter, Germany; 2 Heraeus Medical GmbH, Wehrheim, Germany; 3 Hochschule Fresenius, Wiesbaden, Germany; 4 Department of Trauma Surgery, University Hospital Regensburg, Regensburg, Germany

**Keywords:** Hip fracture, Surgical site infection, Hemiarthroplasty

## Abstract

**Introduction:**

Surgical site infection (SSI) is a major complication following hemiarthroplasty surgery for displaced neck of femur fractures. Our aim is to systematically analyse relevant peer-reviewed studies for recent clinical information on the quantitative risk of surgical site infection (SSI) after hemiarthroplasty (HA) of hip fracture patients and on the factors which influence the SSI rates.

**Methods:**

A comprehensive search of electronic databases (PubMed, Cochrane) was performed for clinical articles published between 2005 and 2023 and systematically reviewed with a defined list of inclusion and exclusion criteria. The methodology was undertaken and reported according to the Preferred Reporting Items for Systematic reviews and Meta-Analyses (PRISMA) statement checklist, while the detailed search strings and study protocol were published in PROSPERO (CRD42023458150). The pooled risks of SSIs were calculated in both primary and subgroup analyses.

**Results:**

The primary analysis showed a pooled superficial SSI rate after hemiarthroplasty of 1.3% (95% confidence interval (CI) 0.71; 2.04) from 17 studies with 29,288 patients and a deep SSI rate of 2.14% (1.87; 2.42) from 29 studies with 192,392 patients. Higher infection rates were observed with longer follow-up periods for deep SSI: pooled rates increased from 1.24% (0.73; 1.87) at 1 month to 2.64% (2.03; 3.31) at 12 months. Additionally, studies using defined criteria for infection diagnosis reported higher rates compared to undefined criteria: pooled deep SSI rates were 2.91% (1.40; 4.92) vs. 0.62% (0.34; 0.96) for defined vs. undefined criteria respectively, and 3.18% (2.23; 4.29) vs. 1.7% (1.44; 1.99) for superficial SSI.

**Conclusions:**

The results of this study demonstrate a substantial SSI risk and a high variability of the infection rates following hemiarthroplasty for hip fracture patients. A standardization of infection criteria and an extended follow-up period are advisable and should be considered in guidelines aimed at improving the standard of care for these patients.

**Supplementary Information:**

The online version contains supplementary material available at 10.1007/s00402-024-05384-5.

## Introduction

Population ageing is an increasing demographic trend which affects many countries across the globe. Associated with ageing is a rise in severe low energy injuries such as falls from a standing position, affecting above all the frail geriatric patient. Such traumas often lead to hip fractures (HF) as a consequence of poor bone quality and severe osteoporosis. The global number of HF is expected to increase from 1.26 million in 1990 to 4.5 million by the year 2050 [1]. In the UK, the health community and orthopaedic surgeons have been prepared for an increasing volume of osteoporotic fractures which may in future overwhelm the medical services and lead to estimated costs between £2–3 billion by 2030 [2]. These calculations do not even include the enormous social costs due to the high mortality and morbidity post-discharge.

Usually, HF treatment requires surgery to repair or replace the fractured hip. Displaced neck of femur fractures are particularly problematic as a consequence of the blood supply disruption to the head of the femur and are therefore typically treated with urgent hemi- or total hip arthroplasty, especially in the elderly population [3]. Given the high level of frailty such patients are even more susceptible to developing some of the hip replacement associated complications including periprosthetic fractures, loosening, dislocations or infections [4]. However, the incidence of these complications in HF populations have been largely reported on basis of single centre experiences or on larger national cohorts in the past and may not mirror the now more stringent contemporary femur fracture treatment recommendations issued by some recent national guidelines [5–7]. In addition, substantial cross-country variations in care for patients presenting with HFs seem to exist [8]. 

Surgical site infections (SSI) have been recognised as one of the most debilitating complications in HF patients. They are associated with higher morbidity and mortality rates, increased length of hospital stays, extensive use of antibiotics, enhanced follow-up, and revision surgeries [9–11]. A particularly severe situation occurs if the infection also affects the deeper joint space and the prosthesis. The burden of SSI on the already high cost of HF has led to the introduction of new surgical methods, bundled payment system, innovative products as well as research with the aim of reducing the rate of SSI in patients after arthroplasty. This rate also remains one of the significant outcomes in clinical and economic studies related to the management of HF.

Given the high degree of variations in care on the one hand and the high impact of SSI in these patients on the other, it is important to gain a comprehensive picture on the overall burden of infections after hemiarthroplasty. Therefore, a qualitative and quantitative systematic review was performed with the objectives of (1) summarizing the available information on SSI rate after hemiarthroplasty in HF patients based on literature from 2005 to July 2023 and (2) identifying factors (e.g. types of SSI, criteria for infection definition, study design, observation periods) that can help explain the variations in these rates.

## Methods

A systematic review of published studies was conducted. The methodology was undertaken and reported according to the PRISMA statement checklist (see Supplementary material) [12]. The study protocol was registered prior to conducting the review with PROSPERO registration number: CRD42023458150.

### Eligibility criteria

Randomized control trials and observational studies that reported rates of SSI after HA following a femoral neck fracture were analysed. The differentiation of superficial SSI and deep SSI was based on the infection criteria provided by the Centers for Disease Control and Prevention’s guidelines which are summarized in the draft guideline [13]. Studies were included and excluded as described in Table 1.

**Table 1:** Inclusion and exclusion criteria

	Included	Excluded
Population	Adults over the age of 18 years undergoing hemiarthroplasty of fractured neck of femur	Patients under the age of 18 years Patients undergoing other and unspecified surgical procedure Patients undergoing revision hemiarthroplasty
Intervention	All relevant interventions related to hip hemiarthroplasty of fractured neck of femur	Other hip procedures and unspecified surgical hip procedures
Comparator	All relevant interventions related to hip hemiarthroplasty of fractured neck of femur	Other hip procedures and unspecified surgical hip procedures
Outcomes	Deep, superficial and combined surgical site infections	Other Outcomes
Studies	Randomized control trials, observational studies (retrospective and prospective)	Meta-analyses, Single-arm studies, Literature reviews, Comments, Editorials, Letters, Lectures, Description and/or video of surgical technique, Retracted publications, Duplicate publications, Case studies, Case reports, Congress abstracts

### Search strategy

Searches were carried out on PubMed (including subsets such as MEDLINE, PMC, etc.) and Cochrane Library. The literature search of PubMed and Cochrane® library was performed on July 13th, 2023. With the aim of identifying recently published literature, the search string was limited to studies published from 2005 onwards. The key search terms were “hip fracture”, “fractured neck of femur”, “hemiarthroplasty”, “PJI”, “SSI”. Detailed search strings were published in the PROSPERO protocol. The search was restricted to human randomized control trials and observational studies published in English.

The abstracts and full tests of the identified records were screened by two individuals using PICO Portal (New York, United States). Studies that passed the screening were managed in the Citavi reference software (Swiss Academic Software, Wädenswil, Switzerland).

### Study selection

The studies were included based on the inclusion criteria outlined in Table S1 (see Supplementary material). Titles and abstracts of studies were initially screened to determine study’s eligibility, then full text of studies reviewed. The studies that fully met the inclusion criteria were included for data extraction.

All records identified by the literature search were retrieved and screened independently by two researchers. The researchers were blinded to each other’s decisions and disagreements between the two researchers were settled by a third reviewer. Detailed methodology of data extract and risk assessment can be found in the Supplementary material.

### Quantitative synthesis

#### Primary analysis

Dichotomous outcomes used in the pooled analysis were reported as number of events per group. In included studies where this is not the case, the number of events was estimated by multiplying the reported risk of events by the total number of patients per group.

Meta-analysis of proportion was carried out to estimate the pooled rate of dichotomous outcomes. It involved the synthesis of a one-dimensional binomial measure known as (weighted) average proportions, estimated by pooling the results (proportions) from various studies and weighted by the inverse of their sampling variances [14]. The heterogeneity of each analysis was assessed using the I2 statistic. The meta-analysis was performed in R using the “metafor” and “meta” packages and the random effects model [14]. 

#### Subgroup analyses

Due to the differences in the follow-up periods reported by the different studies, subgroup analyses were performed for the primary outcomes using follow-up periods of 1-month, 3-months, and 12-months. If the reported rates were outside of these follow-up periods, a rate conversion was performed to the closest follow-up period before the meta-analysis.

A subgroup analysis comparing pooled SSI rates between defined versus undefined SSI was carried out to minimize the effect of missing definition in several papers.

#### Sensitivity analyses

Sensitivity analyses was conducted to evaluate the reliability of the generated findings. Studies deemed to have a high risk of bias, those with an unusually large sample size (characterized as studies exceeding 100% more participants than others in each group), and studies reporting exceptionally extreme outcomes were excluded. The subsequent findings were then compared with the initial analysis to assess the influence of these studies on the overall results.

## Results

### Study selection

**Fig. 1:**
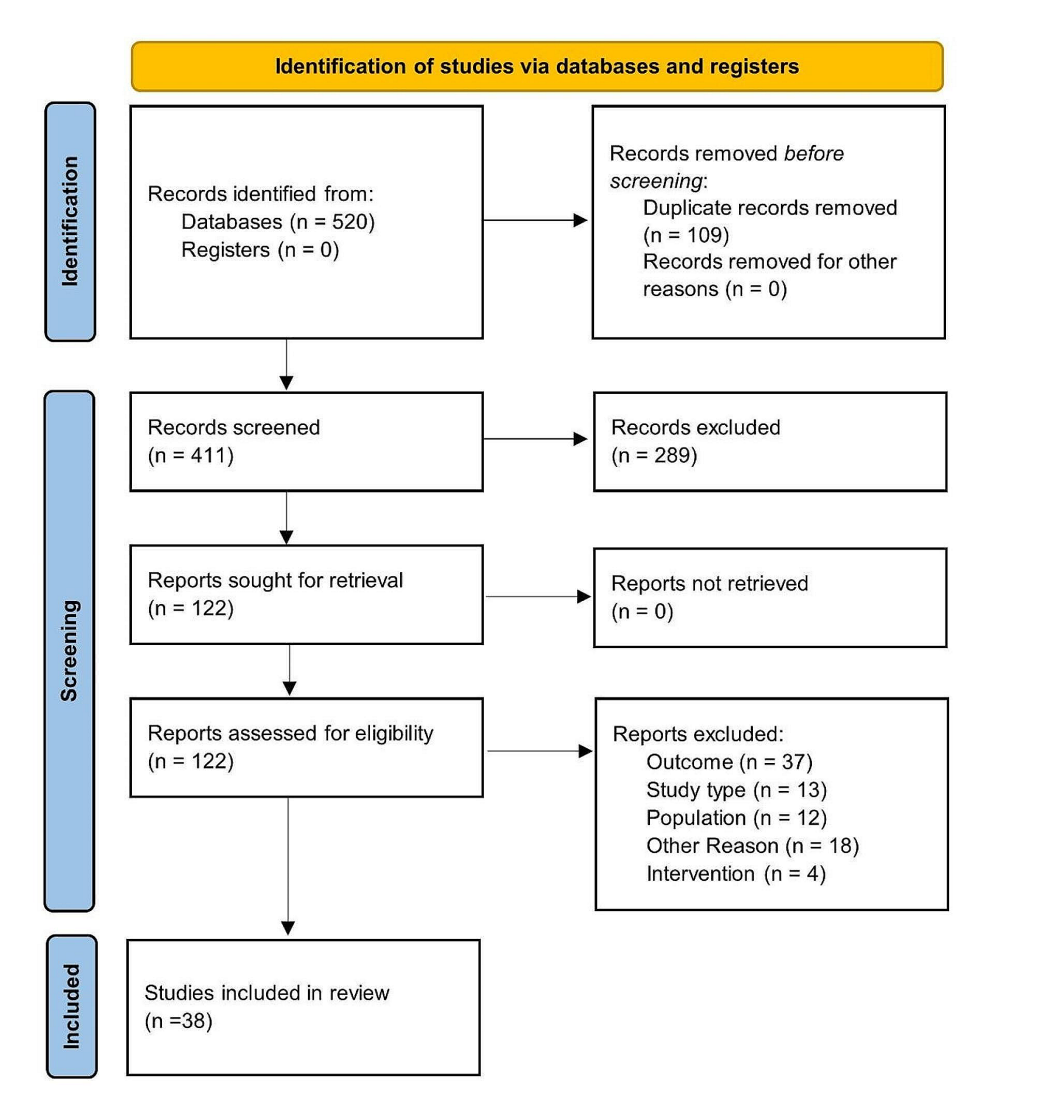
Study selection outlined in a PRISMA flow diagram

The screening process and the steps involved in the selection of included studies are summarized in the PRISMA protocol flow diagram (see Fig. 1). A total of 270 and 141 abstracts were retrieved from the literature search. In total, 122 full text articles were screened, and 38 studies selected for data extraction after application of the eligibility criteria.

### Study characteristics

A summary of the study characteristics including study quality is provided in Table S2 (Supplementary material). The majority of the included studies (71%, 27 studies) were published between 2019 and 2023. The remaining 29% (11 studies) were published between 2005 and 2018. Observational studies (31) reporting retrospective data from databases were most frequent. Two and three of the studies were randomized control trials and clinical studies, respectively. The studies were conducted in a total of 14 countries: China, Finland, France, Germany, Israel, Japan, Netherlands, Norway, Singapore, South Korea, Spain, Turkey, United Kingdom and the United States of America. Most studies (26%,10 studies) were conducted in the United Kingdom, while six and five were reported from the United States and Netherlands, respectively.

In 50% of the studies a definition of SSI was used. Most studies (74%), which reported a definition of infection used the Centres for Disease control and prevention (CDC) definition [13]. Two studies each applied the definition issued by the Infectious Disease Society of America [15], and the UK Health Security Agency [16], while one study used the International Consensus Meeting on Prosthetic Joint Infections (ICMPJI) definition [17, 18].

Included studies reported on superficial SSI, deep SSI, both superficial and deep SSI or combined SSI. Follow-up periods reported by the various studies ranged from 1 month to 10 years. Six of the studies did not report the follow-up period.

### Risk of bias

An overview of the individual study quality per the Newcastle-Ottawa scale can be found in Table S2 (Supplementary material). Eighteen of the included studies had quality ratings of “good” with another eighteen studies rated as “fair”. The remaining two studies were rated as “poor”.

### Primary analysis

#### Superficial surgical site infection

**Fig. 2:**
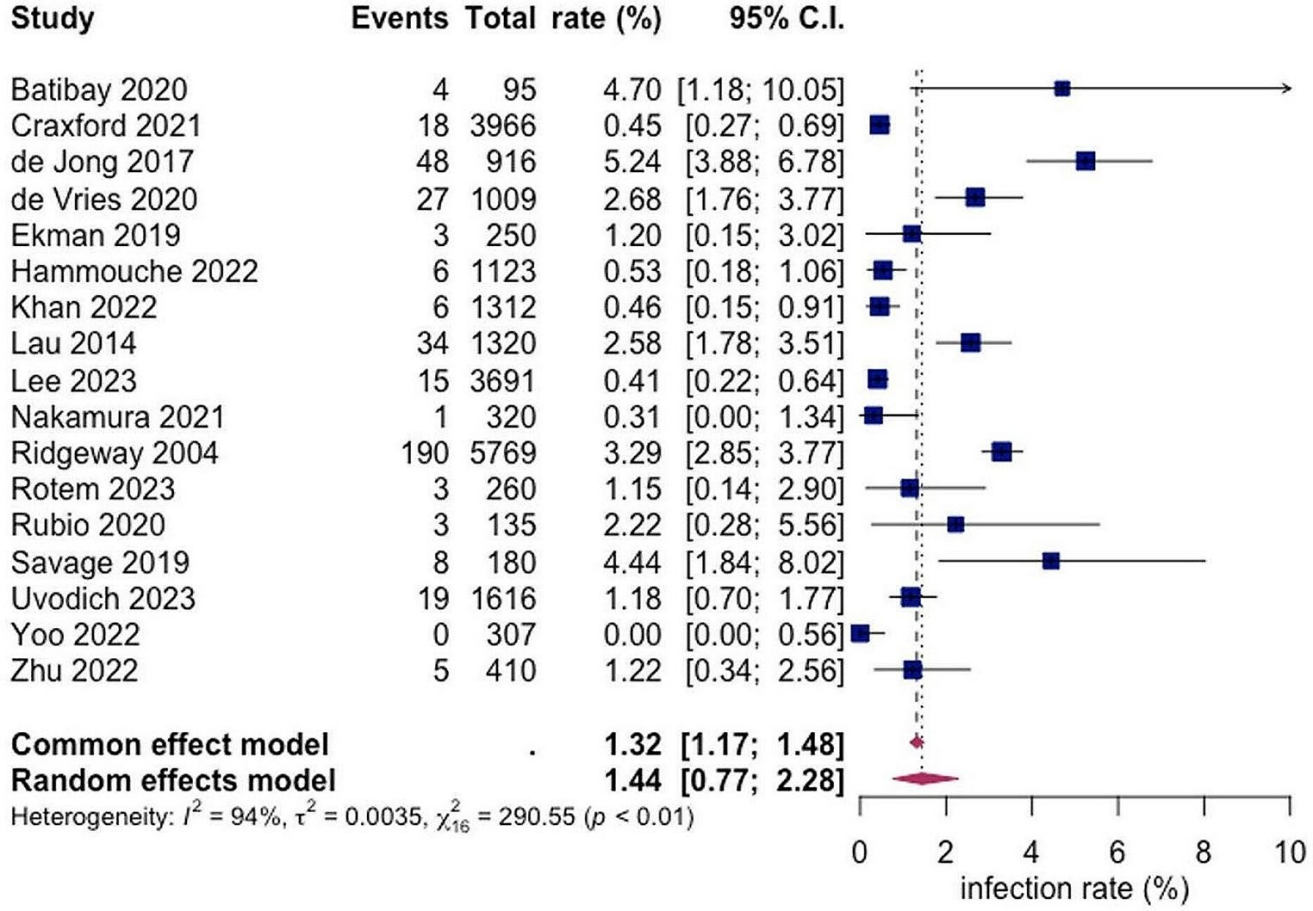
Forest Plot showing the risk of superficial SSI after hemiarthroplasty of hip fracture patients. Overall pooled SSI rate was calculated on basis of 17 studies including 22,679 patients with a value of 1.44% (95% CI 0.77; 2.28). Random effects model was used

A total of 17 studies with a total of 22,679 patients reported on the rate of superficial SSI post hemiarthroplasty with a pooled rate estimated as 1.44% (95% CI 0.77; 2.28) and heterogeneity of 95% (see Fig. 2).

### Deep surgical site infection

**Fig. 3:**
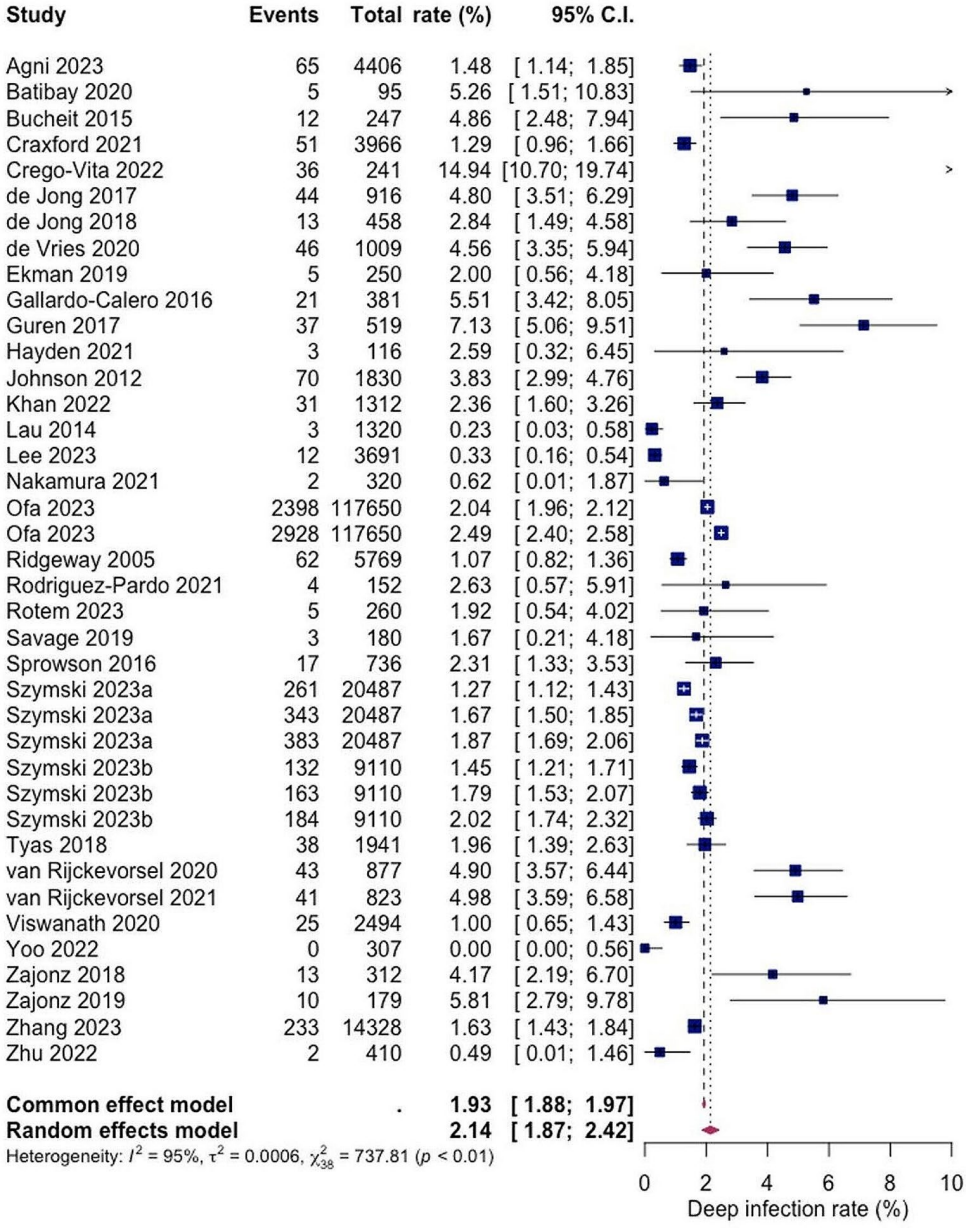
Forest Plot showing the risk of deep SSI after hemiarthroplasty of hip fracture patients. Overall pooled deep SSI rate was calculated on basis 29 studies including 197,092 patients with a value of 2.14% (95% CI 1.87; 2.42). Random effects model was used

A total of 29 studies with a total of 197,092 patients reported on the rate of deep SSI post hemiarthroplasty with a pooled rate estimate of 2.14% (1.87; 2.42) and heterogeneity of 95% (see Fig. 3). Within the included studies, the reported rates of deep SSI varied with the lowest being 0.00% and 14.94% as the highest (see Fig. 3).

### Subgroup analyses

**Fig. 4:**
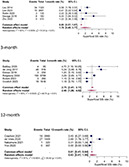
Forest plots showing pooled rates of superficial SSI at different follow-up period; 1-month, 3-month, and 12-month. Random effects model was used

In the subgroup analyses, rates of superficial SSI following HA in hip fracture patients varied across different follow-up periods. For a 1-month follow-up the pooled rate was estimated at 1.78% (0.49%; 3.77%), while for 3-month follow-up an infection rate of 2.46% (1.26%; 4.02%) was calculated. The pooled 12-month rate at 0.63% (0.01; 1.95) was notably lower than the others, this could be because superficial SSI appear within the first week post as they rather involve more virulent bacteria, such as Staphylococcus aureus [19]. Heterogeneity was observed, measuring at 92%, 90% and 91%, for the 1-month, 3-month and 12-month follow-ups, respectively (see Fig. 4).

**Fig. 5:**
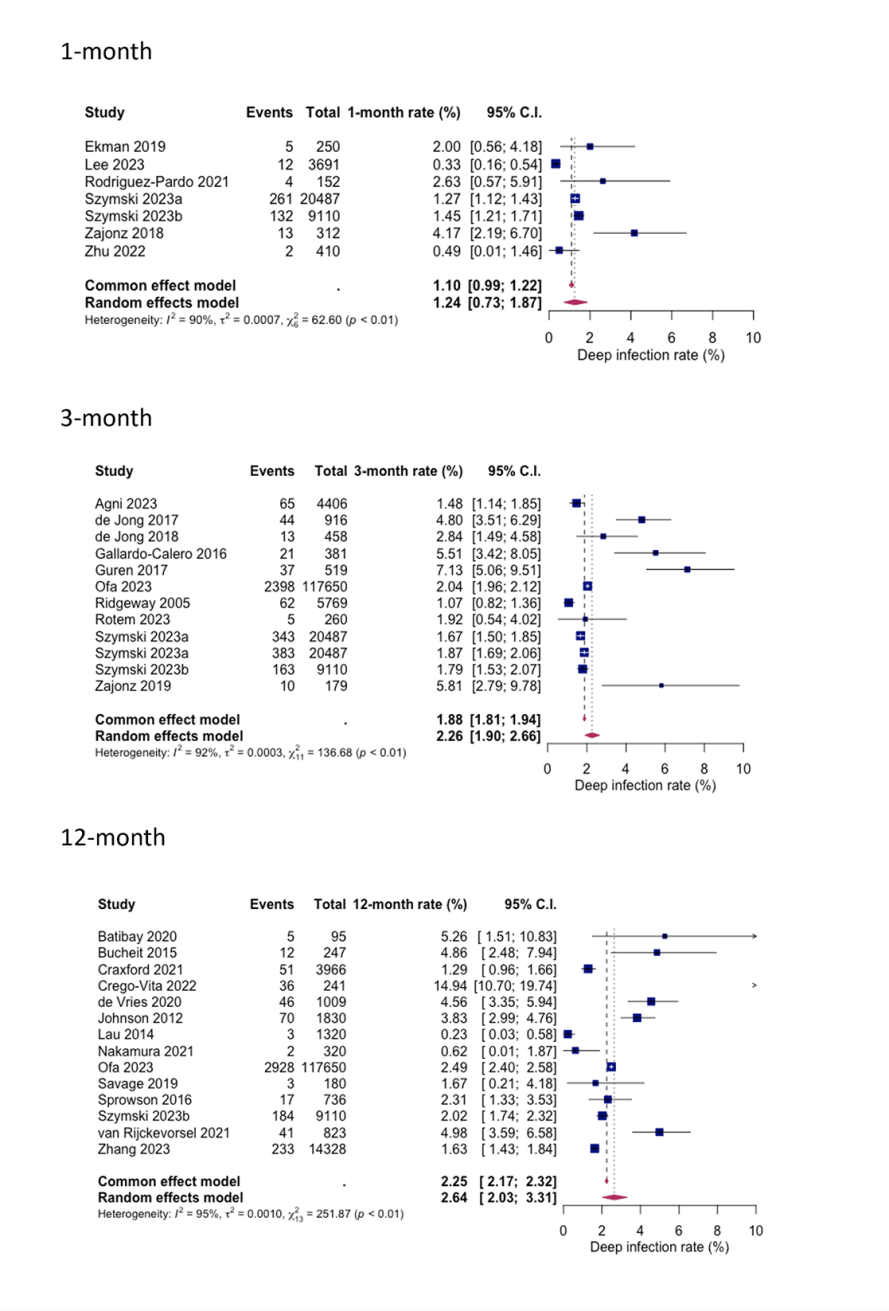
Forest plots showing pooled rates of deep SSI at different follow-up period; 1-month, 3-month, and 12-month. Random effects model was used

Similarly, for deep SSIs, the pooled rates following HA in hip fracture patients were analysed for different follow-up periods. After 1-month 1.24% (0.73%; 1.87%), after 3-months 2.26% (1.90%; 2.66%) and after 12 months 2.64% (2.03%; 3.31%) were identified with a deep SSI. Heterogeneity percentages were observed, measuring at 90%, 93%, and 95% for the 1-month, 3-month, and 12-month follow-ups, respectively (see Fig. 5).

### Defined versus undefined SSI criteria

**Fig. 6:**
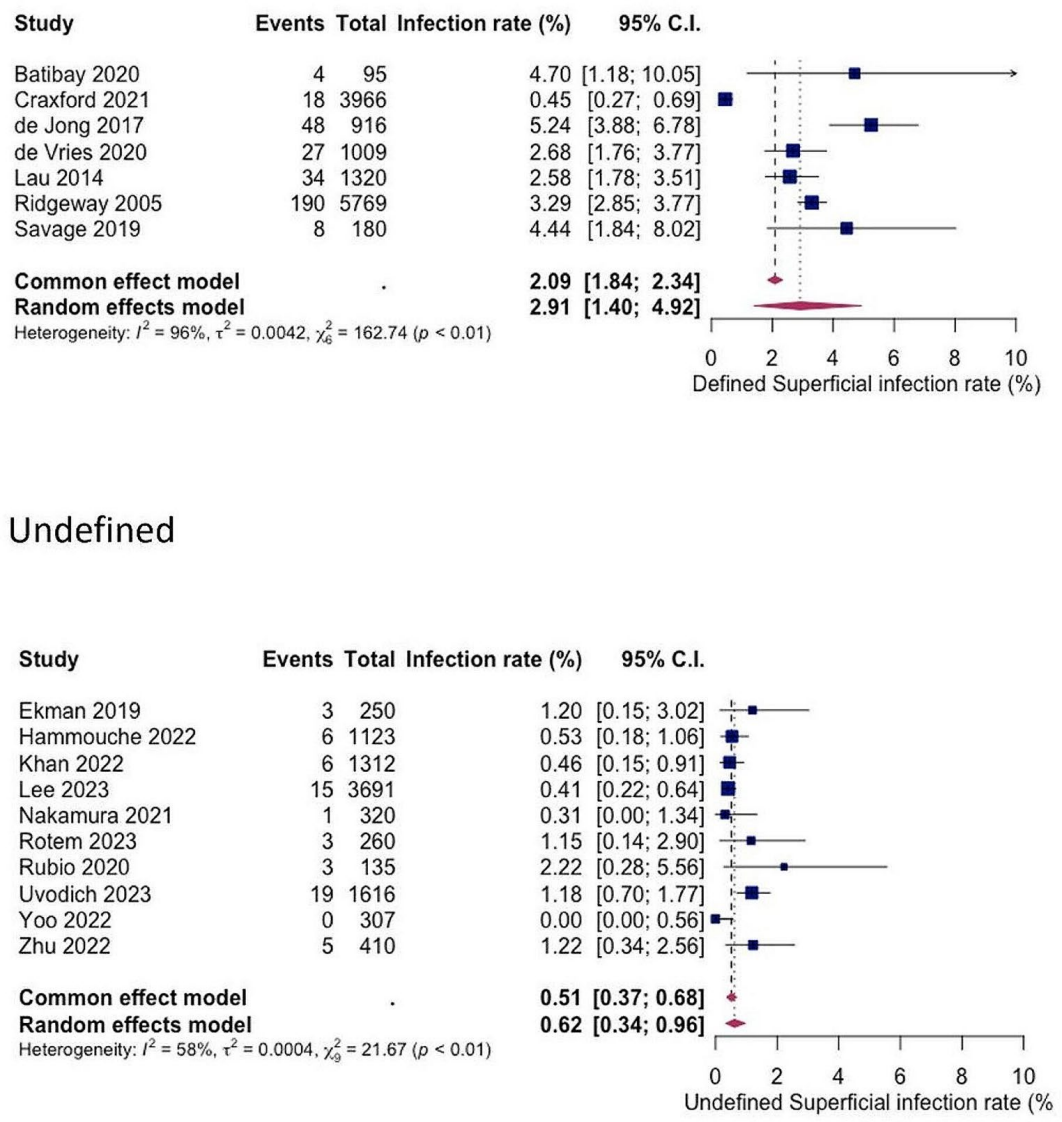
Forest plots showing pooled rates of superficial SSI between studies that used defined and undefined infection criteria. Random effects model was used

**Fig. 7:**
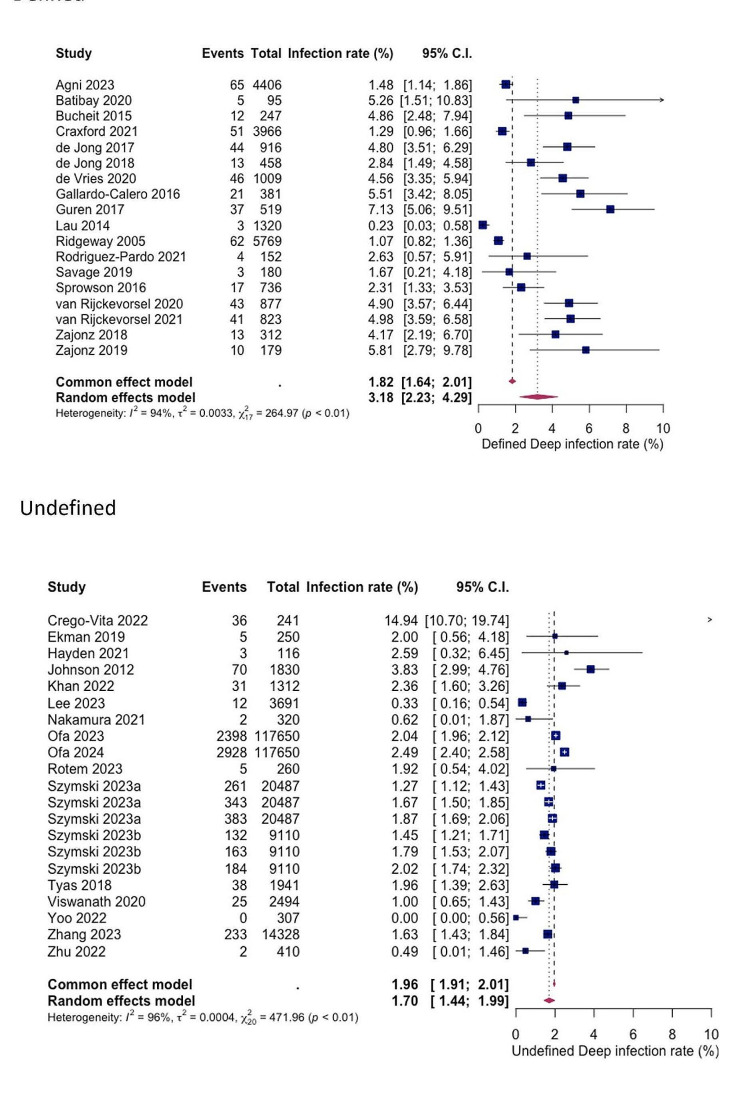
Forest plots showing pooled rates of deep SSI between studies that used defined and undefined infection criteria. Random effects model was used

For both superficial and deep infections, a statistically significant difference in SSI rates was shown between studies with explicitly defined criteria and those with undefined criteria (see Figs. 6 and 7). The pooled superficial SSI rate for defined and undefined criteria were 2.91% (1.40%; 4.92%) and 0.62% (0.34%; 0.96%) respectively, while the pooled deep SSI rates were 3.15% (2.23%; 4.29%) and 1.70% (1.44%; 1.99%) respectively.

### Sensitivity analyses

The impact of outliers, study quality, and sample size on the results of the primary analysis is shown in Figure S1 (see Supplementary material). Overall, the sensitivity analyses showed that the results were mostly robust and consistent.

## Discussion

Our first aim was to assess the overall burden of SSI following HA in hip fracture patients. The results are consistent with previous observations showing that infections are among the most frequent complications in these patients. With a pooled overall rate of 1.3% for superficial SSI and a pooled overall rate of 2.14% for deep SSI with even higher values for extended follow-up periods and defined infection criteria the incidence of infections was higher than usually reported for elective hip arthroplasty procedures [20, 21]. Data from the American Surgical Quality Improvement Program [22] and from the European ECDC Surveillance Atlas for infections [23] report SSI rates following elective primary hip replacement of around 1%. There was also a trend observed towards lower infection rates over the last years which may reflect the implementation of surgical quality improvement programmes in several countries with focus on pre-operative patient optimization, better sterile protocols and surgical techniques or minimizing wound drainage. While these approaches have been effective in primary THA, their impact on hemiarthroplasty is less clear. This is also partially to be expected given the high index of frailty among hip fracture patients and the difficulty to optimize patient risk factors prior to emergency surgery [24]. In fact, a recent population-based cohort study on 74,771 hip fracture patients ≥ 65 years old in Denmark revealed a significantly higher 30-days mortality for those patients who experienced a hospital-treated infection after hip fracture surgery compared to those without infection (hazard ratio = 2.7) [25]. In particular, the risk of developing a subsequent episode of pneumonia or even systemic sepsis was increased several-fold in the infection cohort [25]. 

Our second aim was to analyse factors influencing the SSI rate. Significant variations in infection rates were observed among the studies considered, attributable to differences in (1) infection follow-up periods and (2) infection definition criteria. Notably, extended observation periods beyond the acute phase of one month showed a clear tendency towards higher infection rates. This trend was particularly evident in cases of deep, prosthesis-associated infections, with rates increasing from 1.24% at one month to 2.26% at three months and 2.64% at one year. Given the chronic nature of many implant-related infections, longer follow-up times of at least one year may be necessary to accurately capture delayed infections and ascertain the true incidence of periprosthetic joint infections (PJI). This concern was also highlighted in a recent commentary published in The Lancet, which discussed the observation of a lower-than-assumed overall infection rate during the three-month observation period in a large randomized clinical study involving hemiarthroplasty patients [26]. 

Our observations on the second point reveal significant variations in infection rates depending on the clinical criteria employed for diagnosis. When restricting our analysis to studies utilizing defined and widely accepted infection criteria (such as those outlined by the CDC or UK Health Security Agency) [13, 15], we noted substantially higher infection rates for both types of SSI compared to studies lacking explicit criteria (superficial SSI: defined = 2.91%, undefined = 0.62%; deep SSI: defined = 3.18%, undefined = 1.70%). The CDC definition of SSI, being the earliest and most recognised, classifies infections into superficial incisional, deep incisional, and organ/space categories. Superficial SSI affects only the skin and subcutaneous tissue at the incision site within 30 days post-surgery, while deep SSI involves the fascial and muscle layers within 90 days post-surgery. Organ/space SSI extends beyond these layers to involve bone or joint structures. Despite the lack of a universally accepted definition for PJI, recent evidence suggests that the newly proposed European Bone and Joint Infection Society (EBJIS) classification may offer the highest sensitivity and reliability for diagnosing PJI [27, 28]. Our findings support the notion that unclear infection criteria in clinical practice pose a significant risk of overlooking cases requiring treatment.

Our study has several limitations. The review included observational retrospective studies, which are considered lower in the hierarchy of study qualities. For the prospective studies included, either the average risk of SSI of both control and intervention or the risk reported for only the control was used in the statistical analysis, leading to a potential bias. The review was limited to English language literature. Furthermore, the incidence data were mostly reported only as proportions with no standard deviation around the SSI rates but were only accounted for by meta-analyses of proportion approach. The variation in the duration of follow-up according to the individual objective of the included studies might be responsible for a potential attrition bias used in the pooled estimate. However, this systematic review also has several strengths including a comprehensive search strategy, screening, risk of bias quality assessment, and methodical implementation of the PRISMA.

## Conclusion

The infection rates for both, superficial and deep SSI following HA in hip fracture patients demonstrate a high level of variation within the literature. It was reported that the overall reported infection rates are higher if the follow-up period is extended to up to one year and if established diagnostic criteria for infection definition are used. To enable meaningful analysis and interpretation of studies in the future, the standardized application of established infection criteria for clinical trials is advisable and should be demanded by specialized journals.

## Electronic supplementary material

Below is the link to the electronic supplementary material.

Supplementary Material 1

Supplementary Material 2
