# Supplementary material for: Risk of surgical site infection after hip hemiarthroplasty of femoral neck fractures: a systematic review and meta-analysis
Source: Arch Orthop Trauma Surg. 2024 May 28;144(8):3685–95. doi: 10.1007/s00402-024-05384-5 (PMC11417081; doi:10.1007/s00402-024-05384-5)
Supplement: Supplementary file 1 — Supplementary Material 1 [file 402_2024_5384_MOESM1_ESM.docx]

**Risk of Surgical Site Infection After Hip Hemiarthroplasty of Femoral Neck Fractures: A Systematic Review and Meta-analysis**

**Data extraction**

Data were extracted by two independent researchers from the text, tables, and/or figures of the included studies using a pre-piloted Excel sheet. Discrepancies were resolved through discussion and consensus between the two researchers, or through discussion with a third reviewer.

Data were collected on study design, time frame, population, intervention, comparator groups and follow-up periods as well as study outcomes.

The primary outcomes of interest were deep surgical site infection defined as the number or percentage of patients with infection diagnosed and/or classified as a deep SSI after HA of fractured neck of femur within the study period, Superficial SSI classified as the number or percentage of patients with infection diagnosed and/or classified as a superficial SSI after HA of fractured neck of femur within the study period and combined SSI which was defined as the number or percentage of patients with infection diagnosed and/or classified as a superficial or deep SSI after HA of fractured neck of femur within the study period. No secondary outcomes were considered.

**Risk of bias assessment**

All studies underwent quality appraisal by two researchers using the Newcastle-Ottawa Quality Assessment scale.[1] The assessment scale comprises the domains: selection, comparability and outcomes of the study. Score ranges were grouped into the following three quality levels:

- Good quality: 3 or 4 stars in selection domain AND 1 or 2 stars in comparability domain AND 2 or 3 stars in outcome/exposure domain
- Fair quality: 2 stars in selection domain AND 1 or 2 stars in comparability domain AND 2 or 3 stars in outcomes/exposure domain
- Poor quality: 0 or 1 star in selection domain OR 0 stars in comparability domain OR 0 or 1 stars in outcome /exposure domain

# Tables

# Table S1: PRISMA checklist

| **Section and Topic** | **Item #** | **Checklist item** | **Location where item is reported** |
| --- | --- | --- | --- |
| **TITLE** | | |  |
| Title | 1 | Identify the report as a systematic review. | Title page |
| **ABSTRACT** | | |  |
| Abstract | 2 | See the PRISMA 2020 for Abstracts checklist. | L1 - L30 |
| **INTRODUCTION** | | |  |
| Rationale | 3 | Describe the rationale for the review in the context of existing knowledge. | L34 – L64 |
| Objectives | 4 | Provide an explicit statement of the objective(s) or question(s) the review addresses. | L64 – L 68 |
| **METHODS** | | |  |
| Eligibility criteria | 5 | Specify the inclusion and exclusion criteria for the review and how studies were grouped for the syntheses. | L77 – L85 |
| Information sources | 6 | Specify all databases, registers, websites, organisations, reference lists and other sources searched or consulted to identify studies. Specify the date when each source was last searched or consulted. | L 88 – L89 |
| Search strategy | 7 | Present the full search strategies for all databases, registers and websites, including any filters and limits used. | Prospero protocol |
| Selection process | 8 | Specify the methods used to decide whether a study met the inclusion criteria of the review, including how many reviewers screened each record and each report retrieved, whether they worked independently, and if applicable, details of automation tools used in the process. | L99 – L107 |
| Data collection process | 9 | Specify the methods used to collect data from reports, including how many reviewers collected data from each report, whether they worked independently, any processes for obtaining or confirming data from study investigators, and if applicable, details of automation tools used in the process. | Supplementary material |
| Data items | 10a | List and define all outcomes for which data were sought. Specify whether all results that were compatible with each outcome domain in each study were sought (e.g. for all measures, time points, analyses), and if not, the methods used to decide which results to collect. | Supplementary material |
|  | 10b | List and define all other variables for which data were sought (e.g. participant and intervention characteristics, funding sources). Describe any assumptions made about any missing or unclear information. | Supplementary material |
| Study risk of bias assessment | 11 | Specify the methods used to assess risk of bias in the included studies, including details of the tool(s) used, how many reviewers assessed each study and whether they worked independently, and if applicable, details of automation tools used in the process. | Supplementary material |
| Effect measures | 12 | Specify for each outcome the effect measure(s) (e.g. risk ratio, mean difference) used in the synthesis or presentation of results. | L110 – L113 |
| Synthesis methods | 13a | Describe the processes used to decide which studies were eligible for each synthesis (e.g. tabulating the study intervention characteristics and comparing against the planned groups for each synthesis (item #5)). | L110 – L113 |
|  | 13b | Describe any methods required to prepare the data for presentation or synthesis, such as handling of missing summary statistics, or data conversions. | L110 – L113 |
|  | 13c | Describe any methods used to tabulate or visually display results of individual studies and syntheses. | L114 – L119 |
|  | 13d | Describe any methods used to synthesize results and provide a rationale for the choice(s). If meta-analysis was performed, describe the model(s), method(s) to identify the presence and extent of statistical heterogeneity, and software package(s) used. | L114 – L119 |
|  | 13e | Describe any methods used to explore possible causes of heterogeneity among study results (e.g. subgroup analysis, meta-regression). | L121 – L127 |
|  | 13f | Describe any sensitivity analyses conducted to assess robustness of the synthesized results. | L129 – L134 |
| Reporting bias assessment | 14 | Describe any methods used to assess risk of bias due to missing results in a synthesis (arising from reporting biases). | L129 – L134 |
| Certainty assessment | 15 | Describe any methods used to assess certainty (or confidence) in the body of evidence for an outcome. | L129 – L134 |
| **RESULTS** | | |  |
| Study selection | 16a | Describe the results of the search and selection process, from the number of records identified in the search to the number of studies included in the review, ideally using a flow diagram. | L137 – L144 |
|  | 16b | Cite studies that might appear to meet the inclusion criteria, but which were excluded, and explain why they were excluded. | - |
| Study characteristics | 17 | Cite each included study and present its characteristics. | Supplementary material  L146 - L164 |
| Risk of bias in studies | 18 | Present assessments of risk of bias for each included study. | Supplementary material  L165 - L168 |
| Results of individual studies | 19 | For all outcomes, present, for each study: (a) summary statistics for each group (where appropriate) and (b) an effect estimate and its precision (e.g. confidence/credible interval), ideally using structured tables or plots. | Supplementary material |
| Results of syntheses | 20a | For each synthesis, briefly summarise the characteristics and risk of bias among contributing studies. | L170 - L189 |
|  | 20b | Present results of all statistical syntheses conducted. If meta-analysis was done, present for each the summary estimate and its precision (e.g. confidence/credible interval) and measures of statistical heterogeneity. If comparing groups, describe the direction of the effect. | L170 - L189 |
|  | 20c | Present results of all investigations of possible causes of heterogeneity among study results. | L170 - L189 |
|  | 20d | Present results of all sensitivity analyses conducted to assess the robustness of the synthesized results. | Supplementary material  L225 – L228 |
| Reporting biases | 21 | Present assessments of risk of bias due to missing results (arising from reporting biases) for each synthesis assessed. | - |
| Certainty of evidence | 22 | Present assessments of certainty (or confidence) in the body of evidence for each outcome assessed. | - |
| **DISCUSSION** | | |  |
| Discussion | 23a | Provide a general interpretation of the results in the context of other evidence. | L231- L272 |
|  | 23b | Discuss any limitations of the evidence included in the review. | L274 – L282 |
|  | 23c | Discuss any limitations of the review processes used. | L274 – L282 |
|  | 23d | Discuss implications of the results for practice, policy, and future research. | L287 – L292 |
| **OTHER INFORMATION** | | |  |
| Registration and protocol | 24a | Provide registration information for the review, including register name and registration number, or state that the review was not registered. | L72 – L75 |
|  | 24b | Indicate where the review protocol can be accessed, or state that a protocol was not prepared. | L72 – L75 |
|  | 24c | Describe and explain any amendments to information provided at registration or in the protocol. | - |
| Support | 25 | Describe sources of financial or non-financial support for the review, and the role of the funders or sponsors in the review. | - |
| Competing interests | 26 | Declare any competing interests of review authors. | - |
| Availability of data, code and other materials | 27 | Report which of the following are publicly available and where they can be found: template data collection forms; data extracted from included studies; data used for all analyses; analytic code; any other materials used in the review. | - |

# Table S2 Characteristics of included studies and risk of bias

| Study ID | Study Type |  | Start Year | End Year | Sample Size | Definition of Infection | Type of Infection | Follow up (Months) | Study Quality |
| --- | --- | --- | --- | --- | --- | --- | --- | --- | --- |
| Agni 2023[2] | RCT |  | 2018 | 2021 | 4406 | CDC | Deep | 3months | Good |
| Batibay 2020[3] | Clinical study | Prospective | 2015 | 2016 | 95 | CDC | Deep, Superficial | 12months | Good |
| Bucheit 2015[4] | Observational study | Retrospective | NS | NS | 247 | CDC | Deep | 12months | Fair |
| Craxford 2021[5] | Observational study | Retrospective | 2007 | 2018 | 3966 | CDC | Deep, Superficial | 12months | Fair |
| Crego-Vita 2022[6] | Observational study | Retrospective | 2011 | 2017 | 241 | NS | Deep | 12months | Good |
| de Jong 2017[7] | Observational study | Retrospective | 2011 | 2016 | 916 | CDC | Deep, Superficial | 3months | Fair |
| de Jong 2018[8] | Observational study | Retrospective | 2011 | 2016 | 458 | CDC | Deep | 3months | Good |
| de Vries 2020[9] | Cohort study | Retrospective | 2010 | 2014 | 1009 | CDC | Deep, Superficial | 12months | Good |
| Ekman 2019[10] | Observational study | Retrospective | 2007 | 2008 | 250 | NS | Deep, Superficial | 3months | Fair |
| Gallardo-Calero 2016[11] | Observational study | Retrospective | 2011 | 2013 | 381 | IDSA | Deep | 3months | Fair |
| Gupta 2023[12] | Observational study | Retrospective | 2016 | 2017 | 6169 | CDC | Combined | NS | Fair |
| Guren 2017[13] | Other | Retrospective | 1998 | 2012 | 519 | CDC | Deep | 3months | Fair |
| Hammouche 2022[14] | Cohort study | Retrospective | 2006 | 2014 | 1123 | NS | Superficial | NS | Poor |
| Hayden 2021[15] | Cohort study | Retrospective | 2005 | 2018 | 116 | NS | Deep | NS | Fair |
| Johnson 2012[16] | Observational study | Retrospective | 2001 | 2009 | 1830 | NS | Deep | 12months | Fair |
| Khan 2022[17] | Cohort study | Retrospective | 2003 | 2011 | 1312 | NS | Deep, Superficial | 10years | Poor |
| Lau 2014[18] | Observational study | Retrospective | 2004 | 2012 | 1320 | CDC | Deep, Superficial | 12months | Fair |
| Lee 2023[19] | Observational study | Retrospective | 2005 | 2018 | 3691 | NS | Deep, Superficial | 1month | Good |
| Nakamura 2021[20] | Clinical study | Prospective | 2017 | 2018 | 320 | NS | Deep, Superficial | 6-12months | Fair |
| Ofa 2023[21] | Observational study | Retrospective | 2010 | 2019 | 117650 | NS | Deep | 12months | Good |
| Ridgeway 2005[22] | Observational study | Prospective | 1997 | 2001 | 5769 | CDC | Deep, Superficial | 3months | Fair |
| Rodriguez-Pardo 2021[23] | RCT |  | 2016 | 2018 | 152 | IDSA | Deep | 3months | Good |
| Rotem 2023[24] | Clinical study | Retrospective | 2017 | 2018 | 260 | NS | Deep, Superficial | 3months | Good |
| Rubio 2020[25] | Observational study | Retrospective | 2007 | 2009 | 135 | NS | Superficial | 10years | Fair |
| Savage 2019[26] | Observational study | Retrospective and Prospective | NS | NS | 180 | UK Health Security Agency | Deep, Superficial | 12months | Good |
| Sprowson 2016[27] | Other | Quasi-Randomised | 2008 | 2012 | 736 | UK Health Security Agency | Deep | 12months | Good |
| Szymski 2023a[28] | Observational study | Retrospective | 2012 | 2021 | 20487 | NS | Deep | 60months | Good |
| Szymski 2023b[29] | Observational study | Retrospective | 2012 | 2021 | 9110 | NS | Deep | 60months | Good |
| Tyas 2018[30] | Observational study | Retrospective | 2008 | 2014 | 1941 | NS | Deep | NS | Good |
| Uvodich 2023[31] | Observational study | Retrospective | 2000 | 2019 | 1616 | NS | Superficial | 3months | Fair |
| van Rijckevorsel 2020[32] | Observational study | Retrospective | 2010 | 2016 | 877 | CDC | Deep | NS | Good |
| van Rijckevorsel 2021[33] | Observational study | Retrospective | 2010 | 2016 | 823 | CDC | Deep | 12months | Good |
| Viswanath 2020[34] | Observational study | Retrospective | NS | NS | 2494 | NS | Deep | NS | Fair |
| Yoo 2022[35] | Cohort study | Retrospective | 2015 | 2019 | 307 | NS | Deep, Superficial | 22months | Fair |
| Zajonz 2018[36] | Observational study | Retrospective | 2010 | 2015 | 312 | CDC | Deep | 1month | Fair |
| **Zajonz 2019[37]** | Observational study | Retrospective | 2012 | 2014 | 179 | ICMPJI | Deep | 2months | Fair |
| Zhang 2023[38] | Observational study | Retrospective | 2010 | 2020 | 14328 | NS | Deep | 24months | Good |
| Zhu 2022[39] | Observational study | Retrospective | 2018 | 2019 | 410 | NS | Deep, Superficial | 1month | Good |

N, Number; NS, not stated

# Figures


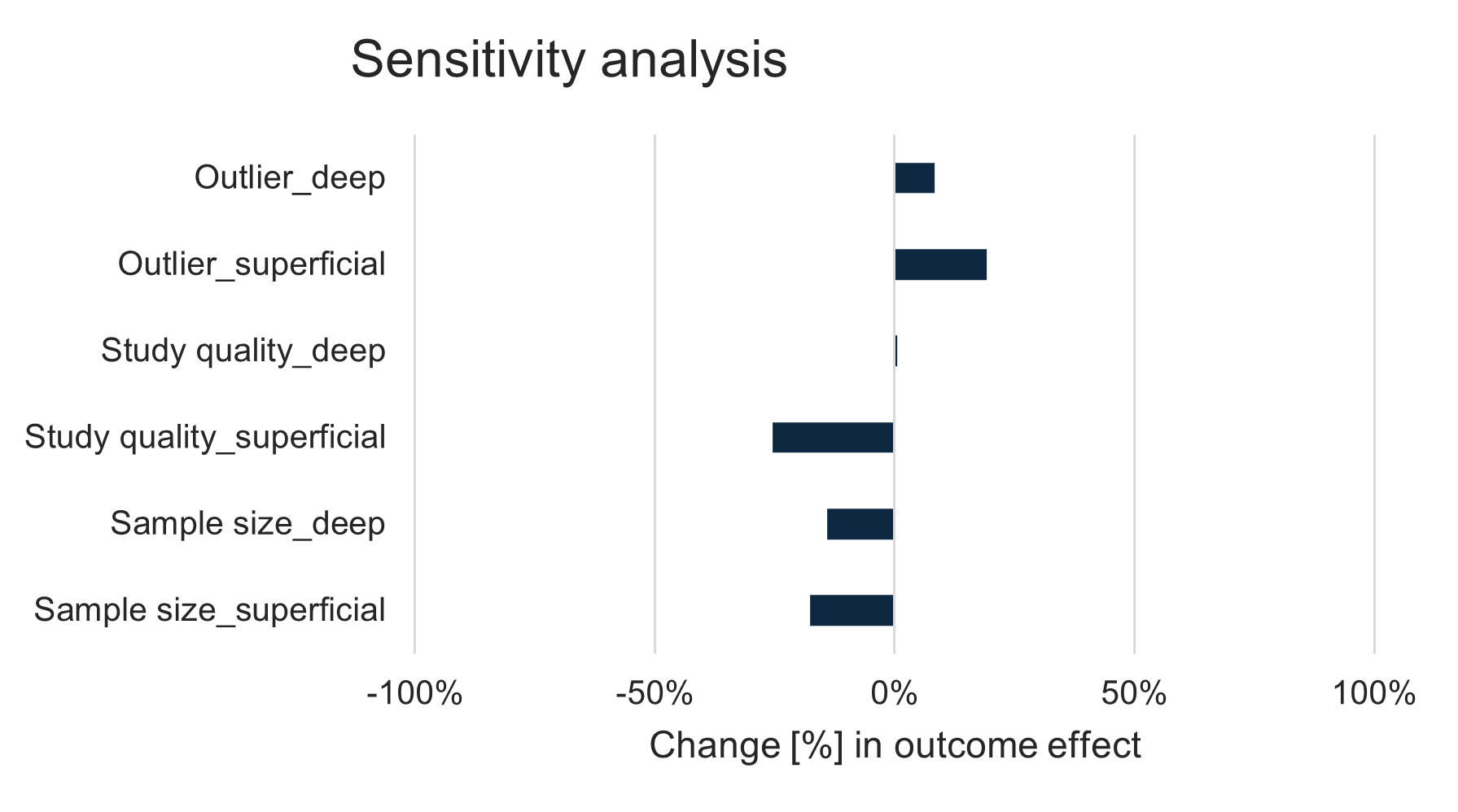


Fig S1: Sensitivity analysis results

References

1. Ottawa Hospital Research Institute The Newcastle-Ottawa Scale (NOS) for assessing the quality of nonrandomised studies in meta-analyses. https://www.ohri.ca/programs/clinical_epidemiology/oxford.asp. Accessed 09 Feb 2024

2. Agni NR, Costa ML, Achten J et al. (2023) High-dose dual-antibiotic loaded cement for hip hemiarthroplasty in the UK (WHiTE 8): a randomised controlled trial. Lancet 402:196–202. https://doi.org/10.1016/S0140-6736(23)00962-5

3. Batibay SG, Soylemez S, Türkmen İ et al. (2020) The effectiveness of preoperative colon cleansing on post-operative surgical site infection after hip hemiarthroplasty. Eur J Trauma Emerg Surg 46:1071–1076. https://doi.org/10.1007/s00068-019-01125-1

4. Buchheit J, Uhring J, Sergent P et al. (2015) Can preoperative CRP levels predict infections of bipolar hemiarthroplasty performed for femoral neck fracture? A retrospective, multicenter study. Eur J Orthop Surg Traumatol 25:117–121. https://doi.org/10.1007/s00590-014-1449-5

5. Craxford S, Marson BA, Nightingale J et al. (2021) Deep infection after hip hemiarthroplasty: risk factors for infection and outcome of treatments. Bone Jt Open 2:958–965. https://doi.org/10.1302/2633-1462.211.BJO-2021-0128.R1

6. Crego-Vita D, Aedo-Martín D, Garcia-Cañas R et al. (2022) Periprosthetic joint infections in femoral neck fracture patients treated with hemiarthroplasty - should we use antibiotic-loaded bone cement? World J Orthop 13:150–159. https://doi.org/10.5312/wjo.v13.i2.150

7. Jong L de, Klem TMAL, Kuijper TM et al. (2017) Factors affecting the rate of surgical site infection in patients after hemiarthroplasty of the hip following a fracture of the neck of the femur. Bone Joint J 99-B:1088–1094. https://doi.org/10.1302/0301-620X.99B8.BJJ-2016-1119.R1

8. Jong L de, Klem TMAL, Kuijper TM et al. (2018) The minimally invasive anterolateral approach versus the traditional anterolateral approach (Watson-Jones) for hip hemiarthroplasty after a femoral neck fracture: an analysis of clinical outcomes. Int Orthop 42:1943–1948. https://doi.org/10.1007/s00264-017-3756-z

9. Vries EN de, Gardenbroek TJ, Ammerlaan H et al. (2020) The optimal approach in hip hemiarthroplasty: a cohort of 1009 patients. Eur J Orthop Surg Traumatol 30:569–573. https://doi.org/10.1007/s00590-019-02610-4

10. Ekman E, Nurmi H, Reito A et al. (2019) Complications following 250 cemented modular hip hemiarthroplasties. Scand J Surg 108:321–328. https://doi.org/10.1177/1457496918812226

11. Gallardo-Calero I, Larrainzar-Coghen T, Rodriguez-Pardo D et al. (2016) Increased infection risk after hip hemiarthroplasty in institutionalized patients with proximal femur fracture. Injury 47:872–876. https://doi.org/10.1016/j.injury.2015.12.032

12. Gupta A, Shin J, Oliver D et al. (2023) Incidence and risk factors for surgical site infection (SSI) after primary hip hemiarthroplasty: an analysis of the ACS-NSQIP hip fracture procedure targeted database. Arthroplasty 5:1. https://doi.org/10.1186/s42836-022-00155-2

13. Guren E, Figved W, Frihagen F et al. (2017) Prosthetic joint infection-a devastating complication of hemiarthroplasty for hip fracture. Acta Orthop 88:383–389. https://doi.org/10.1080/17453674.2017.1301009

14. Hammouche SA, Phillips J, Massoumi A et al. (2022) Implant and patient survival rates using Exeter Trauma Stem hemiarthroplasty in fracture neck of femur patients: The largest study to date. Injury 53:2199–2206. https://doi.org/10.1016/j.injury.2022.03.019

15. Hayden BL, Varady NH, Abdeen A et al. (2021) No Difference Between Hemiarthroplasty and Total Hip Arthroplasty in the Treatment of Pathologic Femoral Neck Fractures. J Arthroplasty 36:3662–3666. https://doi.org/10.1016/j.arth.2021.06.015

16. Johnson B, Starks I, Bancroft G et al. (2012) The effect of care bundle development on surgical site infection after hemiarthroplasty: an 8-year review. J Trauma Acute Care Surg 72:1375–1379. https://doi.org/10.1097/TA.0b013e318245267c

17. Khan SK, Tyas B, Shenfine A et al. (2022) Reoperation and revision rates at ten years after 1,312 cemented Thompson's hemiarthroplasties : any need to change to a different implant? Bone Jt Open 3:710–715. https://doi.org/10.1302/2633-1462.39.BJO-2022-0084

18. Lau ACK, Neo GH, Lee HC (2014) Risk factors of surgical site infections in hip hemiarthroplasty: a single-institution experience over nine years. Singapore Med J 55:535–538. https://doi.org/10.11622/smedj.2014137

19. Lee R, Lee D, Heyer JH et al. (2023) Hip hemiarthroplasty for the treatment of femoral neck fractures in dialysis patients. Hip Int 33:338–344. https://doi.org/10.1177/11207000211028151

20. Nakamura T, Yamakawa T, Hori J et al. (2021) Conjoined tendon preserving posterior approach in hemiarthroplasty for femoral neck fractures: A prospective multicenter clinical study of 322 patients. J Orthop Surg (Hong Kong) 29:23094990211063963. https://doi.org/10.1177/23094990211063963

21. Ofa SA, Lupica GM, Lee OC et al. (2023) Complications following total hip arthroplasty and hemiarthroplasty for femoral neck fractures in patients with a history of lumbar spinal fusion. Arch Orthop Trauma Surg 143:817–827. https://doi.org/10.1007/s00402-021-04158-7

22. Ridgeway S, Wilson J, Charlet A et al. (2005) Infection of the surgical site after arthroplasty of the hip. J Bone Joint Surg Br 87:844–850. https://doi.org/10.1302/0301-620X.87B6.15121

23. Rodríguez-Pardo D, Del Toro MD, Guío-Carrión L et al. (2021) Role of asymptomatic bacteriuria on early periprosthetic joint infection after hip hemiarthroplasty. BARIFER randomized clinical trial. Eur J Clin Microbiol Infect Dis 40:2411–2419. https://doi.org/10.1007/s10096-021-04241-2

24. Rotem G, Lachnish J, Gazit T et al. (2023) Hemiarthroplasty for Hip Fractures: Posterior or Direct Lateral Approach? Advantages and Disadvantages. Isr Med Assoc J 25:91–95

25. Rubio I, Bellostas L, García-Rey E (2020) Radiological subsidence and acetabular erosion after tapered uncemented hemiarthroplasty in femoral neck fractures a 10- to 13- year follow-up study. Injury 51 Suppl 1:S37-S41. https://doi.org/10.1016/j.injury.2020.02.012

26. Savage P, McCormick M, Al-Dadah O (2019) Arthroplasty infection rates in fractured neck of femur: single vs dual antibiotic cement. Ann R Coll Surg Engl 101:514–518. https://doi.org/10.1308/rcsann.2019.0054

27. Sprowson AP, Jensen C, Chambers S et al. (2016) The use of high-dose dual-impregnated antibiotic-laden cement with hemiarthroplasty for the treatment of a fracture of the hip: The Fractured Hip Infection trial. Bone Joint J 98-B:1534–1541. https://doi.org/10.1302/0301-620X.98B11.34693

28. Szymski D, Walter N, Krull P et al. (2023) The Prophylactic Effect of Single vs. Dual Antibiotic-Loaded Bone Cement against Periprosthetic Joint Infection Following Hip Arthroplasty for Femoral Neck Fracture: An Analysis of the German Arthroplasty Registry. Antibiotics (Basel) 12. https://doi.org/10.3390/antibiotics12040732

29. Szymski D, Walter N, Krull P et al. (2023) Infection after intracapsular femoral neck fracture - does antibiotic-loaded bone cement reduce infection risk after hemiarthroplasty and total hip arthroplasty? Bone Joint Res 12:331–338. https://doi.org/10.1302/2046-3758.125.BJR-2022-0314.R1

30. Tyas B, Marsh M, Oswald T et al. (2018) Antibiotic resistance profiles of deep surgical site infections in hip hemiarthroplasty; comparing low dose single antibiotic versus high dose dual antibiotic impregnated cement. J Bone Jt Infect 3:123–129. https://doi.org/10.7150/jbji.22192

31. Uvodich ME, Dugdale EM, Duong SQ et al. (2023) The Association Between Preoperative International Normalized Ratio Values and Postoperative Outcomes including Mortality in Geriatric Femoral Neck Fractures Treated With Hemiarthroplasty. J Orthop Trauma 37:230–236. https://doi.org/10.1097/BOT.0000000000002557

32. van Rijckevorsel VAJIM, Jong L de, Klem TMAL et al. (2022) Drain versus no drain after hip hemi-arthroplasty for femoral neck fractures; differences in clinical outcomes. Eur J Trauma Emerg Surg 48:1799–1805. https://doi.org/10.1007/s00068-020-01528-5

33. van Rijckevorsel VAJIM, Roukema GR, Klem TMAL et al. (2021) Antithrombotics in Patients With a Femoral Neck Fracture: Evaluating Daily Practice in an Observational Cohort Study. Geriatr Orthop Surg Rehabil 12:21514593211037755. https://doi.org/10.1177/21514593211037755

34. Viswanath A, Malik A, Chan W et al. (2020) Treatment of displaced intracapsular fractures of the femoral neck with total hip arthroplasty or hemiarthroplasty. Bone Joint J 102-B:693–698. https://doi.org/10.1302/0301-620X.102B6.BJJ-2019-1459.R1

35. Yoo J-H, Kwak D, Lee Y et al. (2022) Clinical results of short external rotators preserving posterolateral approach for hemiarthroplasty after femoral neck fractures in elderly patients. Injury 53:1164–1168. https://doi.org/10.1016/j.injury.2021.12.049

36. Zajonz D, Behrens J, Brand AG et al. (2019) Hemiarthroplastik bei Oberschenkelhalsbruch im hohen Alter : Auswirkung der präoperativen Wartezeit und des Operationszeitpunkts auf das Risiko periprothetischer Infektionen (Femoral neck fractures in old age treated with hemiarthroplasty : Effects of preoperative waiting time and timing of surgery on the risk of periprosthetic infections). Orthopade 48:224–231. https://doi.org/10.1007/s00132-018-03664-8

37. Zajonz D, Brand A, Lycke C et al. (2019) Risk factors for early infection following hemiarthroplasty in elderly patients with a femoral neck fracture. Eur J Trauma Emerg Surg 45:207–212. https://doi.org/10.1007/s00068-018-0909-8

38. Zhang Z, Chi J, Xu Z et al. (2023) Outcomes Following Hemiarthroplasty for Femoral Neck Fracture in Patients Who Have Hemiparesis. J Arthroplasty 38:2342-2346.e1. https://doi.org/10.1016/j.arth.2023.05.051

39. Zhu J, Hu H, Deng X et al. (2022) Risk factors analysis and nomogram construction for blood transfusion in elderly patients with femoral neck fractures undergoing hemiarthroplasty. Int Orthop 46:1637–1645. https://doi.org/10.1007/s00264-022-05347-8
