# Supplementary material for: Risk of surgical site infection after hip hemiarthroplasty of femoral neck fractures: a systematic review and meta-analysis
Source: Arch Orthop Trauma Surg. 2024 May 28;144(8):3685–95. doi: 10.1007/s00402-024-05384-5 (PMC11417081; doi:10.1007/s00402-024-05384-5)
Supplement: Supplementary file 2 — Supplementary Material 2 [file 402_2024_5384_MOESM2_ESM.docx]

**Conflict of Interest**

- Ubong Silas and Priscilla Anyimiah serves a consultant and received consultancy fee for conducting the systematic review of literature and carrying out meta-analysis.
- Dr. Christof berberich is an employee of Heraeus Medical GmbH, Germany
- Dr. Dominik Szymski and Prof. Dr. Markus Rupp are orthopaedic surgeons practicing in the University Hospital Regensburg, Germany
